# Morin provides therapeutic effect by attenuating oxidative stress, inflammation, endoplasmic reticulum stress, autophagy, apoptosis, and oxidative DNA damage in testicular toxicity caused by ifosfamide in rats

**DOI:** 10.22038/IJBMS.2023.71702.15580

**Published:** 2023

**Authors:** Fatma Cakmak, Sefa Kucukler, Cihan Gur, Selim Comakli, Mustafa Ileriturk, Fatih Mehmet Kandemir

**Affiliations:** 1 Private Buhara Hospital, Emergency Medicine Clinic, Erzurum, Turkey; 2 Atatürk University, Faculty of Veterinary Medicine, Department of Biochemistry, Erzurum, Turkey; 3 Atatürk University, Faculty of Veterinary Medicine, Department of Pathology, Erzurum, Turkey; 4 Atatürk University, Horasan Vocational College, Department of Animal Science, Erzurum, Turkey; 5 Aksaray University, Faculty of Medicine, Department of Medical Biochemistry, Aksaray, Turkey

**Keywords:** Apoptosis, Ifosfamide, Inflammation, Morin, Oxidative stress, Testicular toxicity

## Abstract

**Objective(s):**

In the present study, it was evaluated whether morin has a protective effect on testicular toxicity caused by ifosfamide (IFOS), which is used in the treatment of various malignancies.

**Materials and Methods:**

For this purpose, 100 or 200 mg/kg morin was given to Sprague Dawley rats for 2 days, and a single dose (500 mg/kg) IFOS was administered on the 2nd day. At the 24th hr of IFOS administration, animals were decapitated and testicular tissues were taken and the status of oxidative stress, inflammation, endoplasmic reticulum stress (ERS), autophagy, and apoptosis markers were analyzed by biochemical, molecular, and histopathological methods.

**Results:**

According to the data obtained, it was determined that IFOS caused oxidative stress in testicular tissues. It was observed that inflammation, ERS, autophagy, apoptosis, and oxidative DNA damage occurred with oxidative stress. Morin treatment suppressed oxidative stress. Morin showed anti-inflammatory effects by reducing TNF-α and IL-1β protein levels. It also increased the mRNA transcript levels of the ERS marker ATF-6, PERK, IRE1, GRP-78, and CHOP genes, and the apoptosis marker genes Bax, Casp-3, and apaf-1. It up-regulated the anti-apoptotic protein Bcl-2 gene and the cell survival signal AKT-2 gene. Morin caused a decrease in beclin-1 protein levels and showed an anti-autophagic effect. In addition, morin attenuated oxidative DNA damage and decreased 8-OHdG immune-positive cell numbers.

**Conclusion:**

As a result, it was observed that IFOS caused cellular damage by activating various signaling pathways in testicular tissue, while morin exhibited protective properties against this damage.

## Introduction

Although cancer is the second cause of death in the world, (1) death rates are gradually decreasing thanks to advances in medicine and modern technology. Among these developments, the discovery of new chemotherapeutics has an important place (2). Ifosfamide (IFOS) is one of the chemotherapeutic agents with a cytotoxic effect and is widely used in the treatment of various solid and hematological malignancies (1). This agent, which is an analog of cyclophosphamide (3) and is among the alkylating cytostatics, is used as a prodrug and shows its pharmacological effects with its metabolites synthesized during reactions involving cytochrome p450 enzymes (4). These metabolites are phosphoramide mustard derivatives and acrolein (5). Acrolein is a highly electrophilic compound. Phosphoramide mustard derivatives containing reactive alkyl groups alkylate the nucleophilic groups of DNA, causing cross-linking in DNA (6) and causing apoptosis in damaged cells (5). IFOS is also metabolized to chloroacetaldehyde (7). Chloroacetaldehyde causes depletion of GSH stores and decreases ATP levels due to the suppression of complex I in the mitochondrial respiratory chain (6). This causes the formation of superoxide and the conversion of superoxide into hydrogen peroxide, causing damage to mitochondrial proteins, lipids, and DNA. As a result, apoptosis occurs in cells. With these mechanisms, while IFOS suppresses the proliferation of tumor cells, it also causes serious damage to other tissues. Toxicity occurs in approximately 20% of patients. Among these toxicities, hemorrhagic cystitis, nephropathy, encephalopathy, neurotoxicity, and cardiac toxicity are the major known side effects of IFOS (1, 6). On the other hand, there is insufficient information about the possible effects of IFOS on testicular tissue. Increased levels of reactive oxygen species (ROS) can cause testicular toxicity (8, 9). It is thought that IFOS may damage testicular tissues due to the decrease in GSH stores and increased ROS levels. Providing protection against all these side effects will both remove the limitation in the use of IFOS and increase the quality of life of the patients.

Recently, the protective effects of flavonoids against the side effects of chemotherapeutics have been intensively studied. Studies have shown that compounds with anti-oxidant properties such as flavonoids can alleviate the toxic effect of chemotherapeutic agents that trigger ROS production (10-12). Flavonoids are natural polyphenolic compounds found in many fruits and vegetables (13). In addition to their anti-oxidant properties, they are known to have various pharmacological properties such as anti-inflammatory, anti-autophagic, and anti-apoptotic (14, 15). Due to the remarkable role of oxidative stress in male infertility and the disadvantages of synthetic drugs such as treatment failure, side effects, and high costs, researchers’ interest in natural anti-oxidants has increased. Morin isolated from members of the *Moraceae* family (16) is a natural flavonoid with anti-oxidant, anti-inflammatory, antidiabetic, anti-carcinogenic, neuro-protective, and anti-proliferative effects (17). Thanks to these properties, it has been reported in various studies that morin has a protective effect against testicular toxicity and significantly improves fertility (16, 18). Morin has been reported to modulate the Nrf2 pathway in a study (19). In another study, it was reported that by suppressing ROS production, it reduced the release of apoptotic factors from mitochondria and prevented organ dysfunction (20). On the other hand, in addition to insufficient information on testicular toxicity of IFOS, no study was found on IFOS-induced testicular damage of morin. 

Therefore, in the present study, whether morin has a protective effect on IFOS-induced testicular damage was investigated by histopathological examinations as well as analyses of markers playing a role in oxidative stress, endoplasmic reticulum stress, inflammation, autophagy, and apoptosis pathways.

## Materials and Methods

***Chemicals used in the study***

Ifosfamide (Holoxan) was purchased from Eczacıbaşı (Istanbul, Turkey). All other chemicals, including morin, were obtained from Sigma-Aldrich (St.Louis, MO, USA).

***Characteristics and care conditions of animals used in the study***

The animals used in the study (male Sprague Dawley rats) were obtained from the Experimental Animal Center of Ataturk University (Erzurum, Turkey) and their care and experimentation were done in this center. Before the experiment, the age of the animals was 10-12 weeks and their weight was 220-250 g. Rats had access to standard pellet feed and water *ad libitum*. The environment in which they were kept had a temperature of 24±1 ^°^C and a humidity of 45±5%. Also, animals were subjected to a 12-hour light and 12-hour dark cycle. Ethics committee approval was obtained from Atatürk University Animal Experiments Local Ethics Committee for the study (Protocol No: 2022/8/159).

***Design of the study***

Thirty-five rats in the study were randomly divided into 5 groups (Control, Morin, IFOS, IFOS+Morin 100, and IFOS+Morin 200). There were 7 animals in each group. IFOS and Morin doses selected for applications were determined according to previous studies (3, 21). The animals in the control group were given saline orally for 2 days, and on the 2nd day, additional saline was given intraperitoneally. Animals in the morin group were given orally 200 mg/kg of morin. The animals in the IFOS group were given saline orally for 2 days and 500 mg/kg body weight IFOS was administered on the 2nd day. Animals in the IFOS+Morin 100 group were given 100 mg/kg body weight morin for 2 days, and 500 mg/kg IFOS was administered intraperitoneally on the 2nd day only. Animals in the IFOS+Morin 200 group were given 200 mg/kg body weight morin for 2 days, and 500 mg/kg IFOS was administered intraperitoneally on the 2nd day only. 

The animals were decapitated on the 3rd day under mild sevoflurane anesthesia. Afterward, testicular tissues were quickly removed, one of which was placed in 10% formalin solution for histopathological analysis, and the other was stored at -80 ^°^C until processing for biochemical and molecular analysis.

***Biochemical analyses of oxidative stress markers in testicular tissue***

At this stage, testicular tissues taken from rats were pulverized in liquid nitrogen by means of a tissue lyser (Tissue Lyser II, Qiagen, Netherlands) and then homogenized by diluting 1:10 (w/v) in 1.15% KCl buffer. Homogenates were centrifuged at 3500 RPM for 15 min (for MDA, SOD, and CAT analyses) or 20 min at 10000 RPM (for GSH and GPx analyses). In the supernatants obtained, MDA analyses were performed by Placer, Cushman, and Johnson’s method (22), GSH by Sedlak and Lindsay’s (23) method, CAT by Aebi’s (24) method, GPx by Matkovics’s (25) method, and SOD Sun, Oberley, and Li’s (26) method. In addition, the total protein concentrations of the supernatants were determined by Lowry’s (27) method.

***Total RNA isolation and cDNA synthesis in testis tissue***

Total RNA isolation from the testicular tissues of rats was performed with QIAzol Lysis Reagent (79306; Qiagen). The applications were carried out in strict accordance with the instructions given by the manufacturer. The concentrations of RNAs obtained at the end of the isolation stages were measured in the NanoDrop (BioTek Epoch) device. After RNA concentrations of all groups were equalized at 1000 ng/µl, RNAs were converted into double-stranded cDNA with the iScript cDNA Synthesis Kit (Bio-Rad).

***RT-PCR analysis***

Primer sequences of genes (β-actin was used as internal control) analyzed by the RT-PCR method are presented in Table 1. Primers were designed in the Oligo 6.0 primer design program. In the RT-PCR step, the mixture was prepared with QuaniTect SYBR Green PCR Master Mix (204143; Qiagen), cDNAs, and reverse and forward primers of related genes, according to the manufacturer’s instructions. Then, the reaction was started in the Rotor-Gene Q (Qiagen) device in accordance with the instructions given by the manufacturer. At the end of the procedure, mRNA transcript levels were calculated using the 2^-deltadeltaCT^ method with the CT values obtained from the device (28).

***Western blot analysis of testicular tissues***

In the western blot method, firstly, total protein isolation was performed from testicular tissues using RIPA lysis buffer (Santa Cruz Biotechnology) containing protease inhibitors. Concentrations of isolated proteins were analyzed with the PierceTM BCA Protein Assay Kit (Rockford, IL, USA). Then Laemmli buffer was added to the samples and sodium dodecyl sulfate polyacrylamide gel electrophoresis (SDS-PAGE) was performed. After electrophoresis, the proteins were transferred to the polyvinylidene fluoride (PVDF) membrane by blotting. Membranes of all groups were then blocked with tris-buffered saline containing 5% bovine serum albumin (BSA) and 0.1% Tween 20 for 90 min. At the end of the incubation period, the membranes were incubated with Beclin-1, TNF-α, IL-1β, and β-actin monoclonal antibodies overnight. The next day, after removing the antibodies, the membranes were washed with PBST and then incubated with anti-mouse IgG secondary antibody (1:2000 dilution). At the end of the incubation, protein bands were visualized in the presence of Western ECL Substrate (Bio-Rad, Hercules, USA) with the help of Biorad Gel Doc XR+Imaging System (Bio-Rad, Hercules, USA). Densitometric analysis of blots was performed using the ImageLab program (Bio-Rad, Hercules, USA).

***Histopathological examination***

The rat testis tissues were embedded in paraffin after being fixed in 10% buffered formaldehyde and processed in alcohol-xylene series to assess the histopathologic changes. Then, using a microtome, thin sections of about 4 μm were taken. To evaluate for possible histopathologic changes under a light microscope, sections were deparaffinized, rehydrated using descending alcohols, and stained with the hematoxylin and eosin method. After that, photomicrographs were captured after the slides were examined under a light microscope.

***Immunohistochemical analysis***

Polylysine-coated slides were used to mount the tissue sections. Serial 4 μm thick paraffin-embedded sections were deparaffinized, rehydrated, and kept for 5 min at room temperature in distilled water. The sections were immersed in an antigen retrieval solution (citrate buffer, pH: 6.0) and boiled for 10 min in a microwave, and then cooled to room temperature for 20 min. Sections were washed several times with PBS and treated for 10 min with 3% hydrogen peroxide to quench endogenous peroxidase activity. A protein block was used to decrease nonspecific staining. Afterward, the primary antibody against 8-OHdG (sc-66036, 1:100; Santa Cruz Biotechnology) was added to sections and kept for 60 min, then rinsed in PBS and incubated with a secondary antibody (biotinylated goat anti-polyvalent for 20 min and streptavidin peroxidase for 20 min at room temperature). The sections were counterstained with hematoxylin after being treated with liquid diaminobenzidine (DAB) for 5 min at room temperature. The immune positivity of the sections was scored as follows: none (-), mild (+), moderate (++), and severe (+++).

***Statistical analyzes***

One-way ANOVA and Tukey’s *post hoc* test were performed in SPSS 20.0 software (IBM) program to evaluate the biochemical analyses statistically. Since histopathological data were not normally distributed, we used the nonparametric Kruskal-Wallis test to analyze differences between the groups. The Mann–Whitney U test was used for the comparison of pairs of groups.

## Results

***Effect of ifosfamide and morin on Nrf-2 and HO-1 genes and oxidative stress markers in testicular tissue***

**Figure 1:**
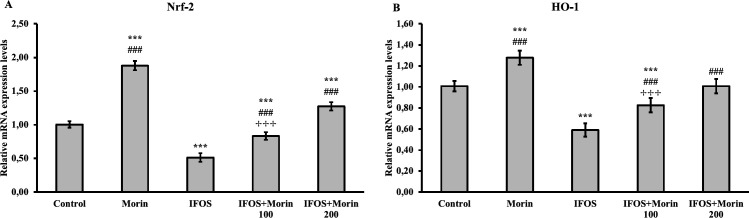
Nrf-2 and HO-1 relative mRNA transcript levels in testis tissues after ifosfamide (IFOS) and morin administration to rats

The mRNA transcript levels of Nrf-2 and HO-1 genes, which are used as markers of oxidative stress in testicular tissue of rats, are presented in Figure 1. The data showed that IFOS can cause oxidative stress by suppressing Nrf-2 and HO-1 genes in testicular tissue. Nrf-2 and HO-1 genes were up-regulated in testicular tissues of rats given morin. In addition, the results revealed that 200 mg/kg morin dose was more effective than 100 mg/kg dose on both Nrf-2 and HO-1 genes.

***Effect of ifosfamide and morin on oxidant and anti-oxidant markers in testicular tissue***

**Figure 2:**
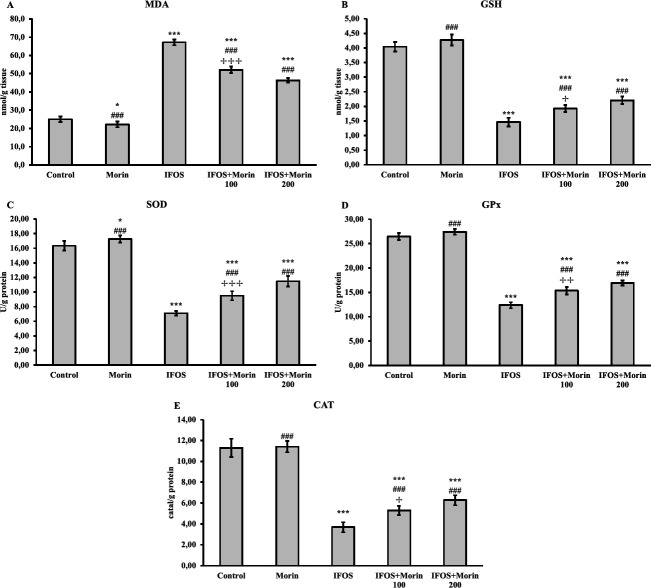
SOD, CAT, and GPx activities and MDA and GSH levels in testicular tissues after IFOS and morin administration to rats

The levels of oxidant marker MDA and anti-oxidant markers GSH, SOD, CAT, and GPx in testicular tissues are summarized in Figure 2. As seen in the figure, IFOS application caused lipid peroxidation in testicular tissues and increased MDA levels. Moreover, it has been determined that IFOS has an inhibitory effect on SOD, CAT, and GPx activities in testicular tissues and depletes GSH stores. It is noteworthy that anti-oxidant enzyme activities are recovered and GSH stores are renewed in the testicular tissues of rats treated with morin. There was also a remarkable decrease in MDA levels after IFOS administration. The data remarkably shows that a high dose is more effective on all markers.

***Effect of ifosfamide and morin on TNF-α and IL-1β in testicular tissue***

**Figure 3:**
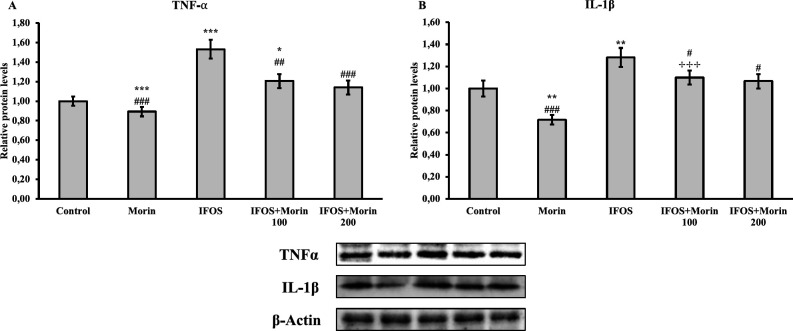
Relative protein levels of TNF-α and IL-1β in testicular tissues after administration of ifosfamide (IFOS) and morin to rats

Evaluation of TNF-α and IL-1β in the testis tissue of rats was done by the Western blot method. As seen in Figure 3, it has been determined that IFOS can cause inflammation by stimulating TNF-α and IL-1β translation in testicular tissue, however, these inflammatory markers can be suppressed by morin. While there was no difference between the doses of TNF-α protein, it was determined that a high dose showed better efficacy on IL-1β.

***Effect of ifosfamide and morin on AKT2 and FOXO1 in testicular tissue***

**Figure 4:**
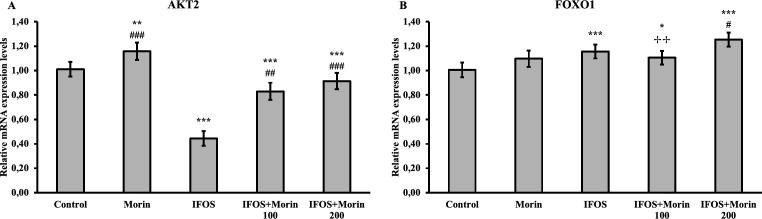
AKT2 and FOXO1 relative mRNA transcript levels in testis tissues after ifosfamide (IFOS) and morin administration to rats

The mRNA transcript levels of the AKT2 and FOXO1 genes analyzed by RT-PCR are summarized in Figure 4. While it was observed that the AKT2 gene was significantly suppressed with IFOS administration, it was determined that FOXO1 increased in an interesting way. The AKT2 gene was significantly increased after morin treatment, but there was no significant difference between doses. When the effects of morin on FOXO1 mRNA transcript levels were evaluated, it was determined that low dose did not make a difference compared to the IFOS group, but FOXO1 was up-regulated in the high dose group.

***Effect of ifosfamide and morin on endoplasmic reticulum stress markers in testicular tissue***

**Figure 5:**
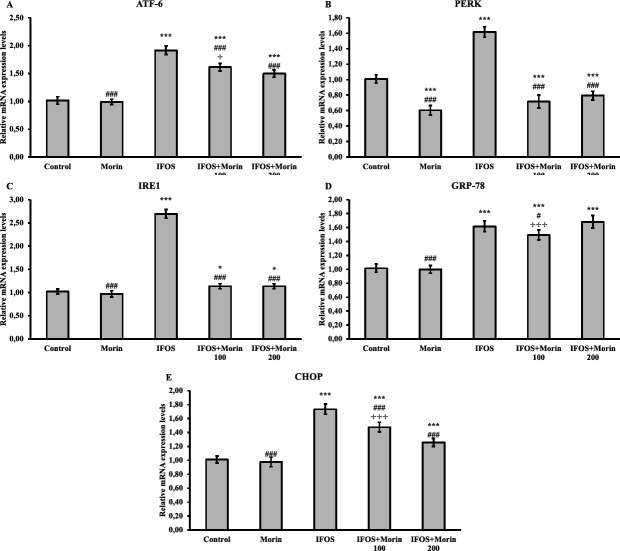
ATF-6, PERK, IRE1, GRP-78, and CHOP relative mRNA transcript levels in testis tissues after ifosfamide (IFOS) and morin administration to rats

ER stress in testis tissue was evaluated by analysis of expression levels of ATF-6, PERK, IRE1, CHOP, and GRP-78 genes (Figure 5). IFOS caused ER stress in testis tissue and induced the expression of related genes. On the other hand, morin treatment suppressed ER stress and decreased the relative mRNA transcript levels of ATF-6, PERK, IRE1, CHOP, and GRP-78. When the evaluation was made between doses in the study, it was determined that the 200 mg/kg dose on ATF-6 and CHOP genes and 100 mg/kg dose on the GRP-78 gene were more effective. There was no significant difference between doses in PERK and IRE1 genes.

***Effect of ifosfamide and morin on apoptosis markers in testicular tissue***

**Figure 6:**
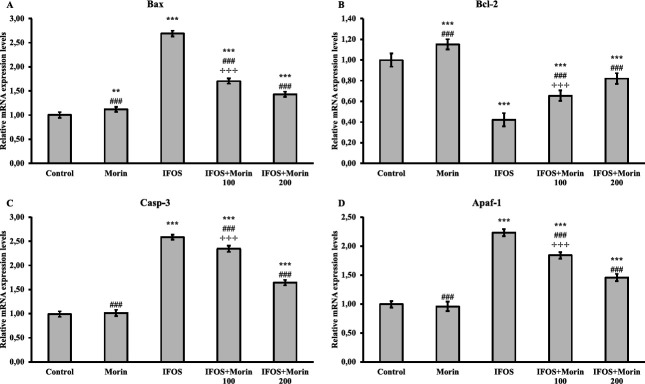
Bax, Bcl-2, Casp-3, and Apaf-1 relative mRNA transcript levels in testis tissues after ifosfamide (IFOS) and morin administration to rats

In the study, the mRNA transcript levels of the apoptotic markers Bax, Casp-3, and Apaf-1 and the anti-apoptotic gene Bcl-2 were analyzed by RT-PCR method to determine the apoptotic state in testicular tissue. The obtained data showed that IFOS up-regulated Bax, Casp-3, and Apaf-1 genes and down-regulated Bcl-2. After morin administration, it was found that Bax, Casp-3, and Apaf-1 were suppressed and Bcl-2 was induced, so that it could protect against IFOS-induced apoptosis. Also, the results showed that high-dose morin may be more effective against apoptosis. All results are given in Figure 6.

***Effect of ifosfamide and morin on Beclin-1, an autophagic indicator in testicular tissue***

**Figure 7:**
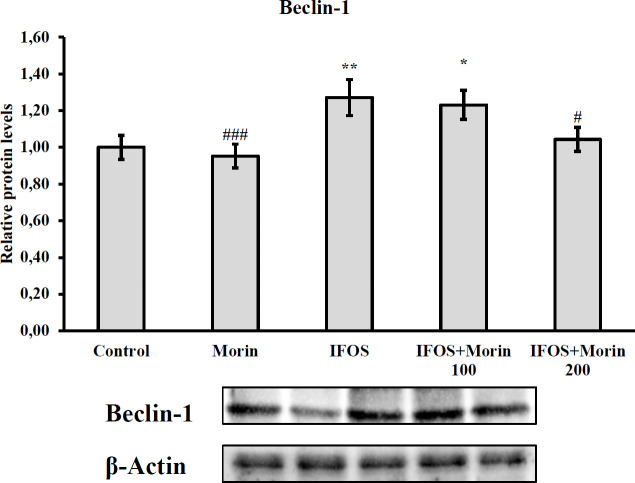
Relative protein levels of Beclin-1 in testicular tissues after administration of ifosfamide (IFOS) and morin to rats

It was determined that Beclin-1, whose relative protein levels are presented in Figure 7, showed a statistically significant increase after IFOS administration. It was observed that 100 mg/kg morin had no effect on IFOS-induced beclin 1, but 200 mg/kg dose reduced the levels of this protein. However, in the data obtained that there is no significant difference between the low-dose and high-dose groups.

***Impacts of morin on histopathological changes in testis tissue***

**Figure 8:**
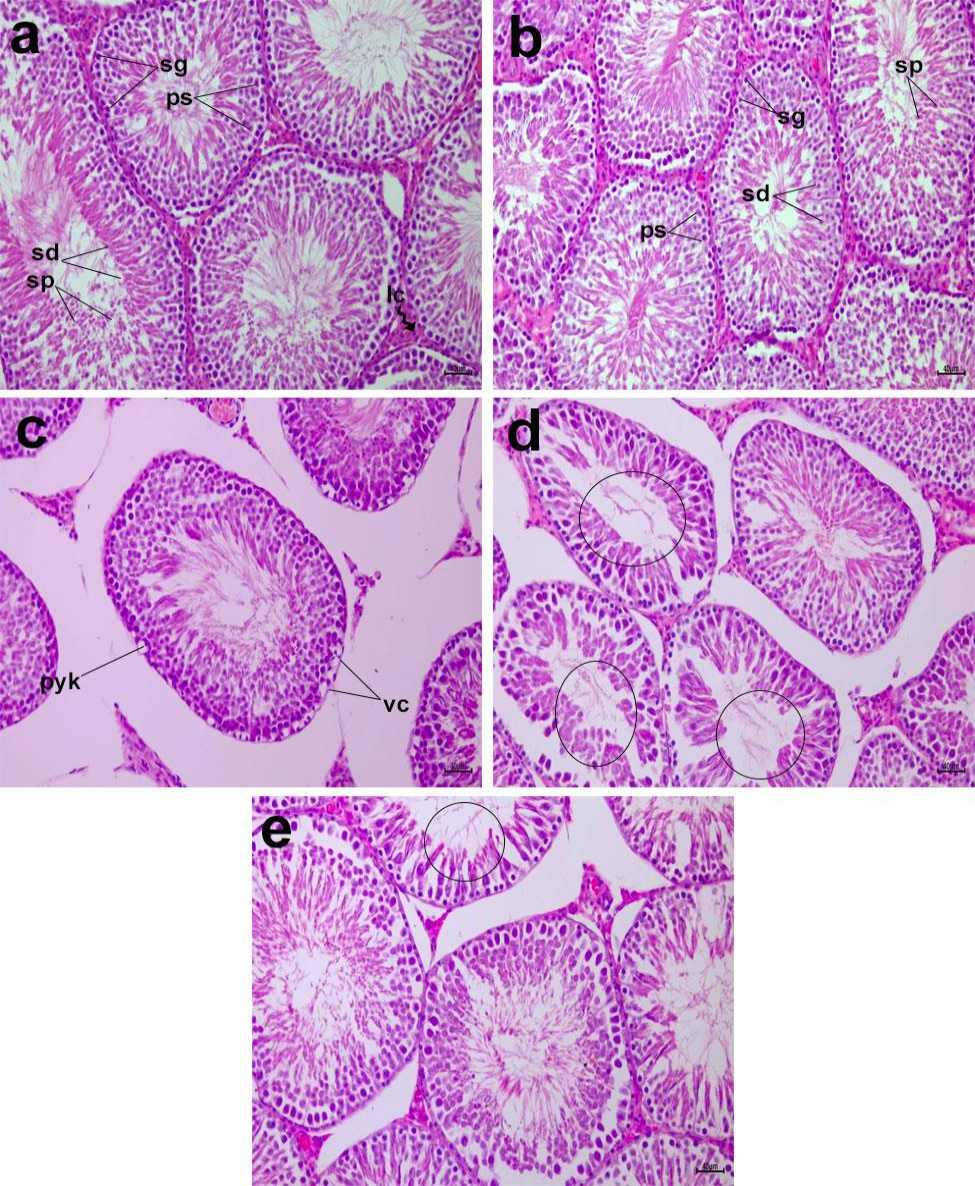
Photomicrographs of a section of testis of rats treated with morin and ifosfamide

Control and morin-treated rats’ testis tissues had normal histology structures for seminiferous tubules (regularly and tightly) and interstitial tissue containing Leydig cells (Figures 8a and 8b). Every tubule contained spermatogonia cells, including primary spermatocytes and early or late spermatids, displaying the entire process of spermatogenesis. The testicular tissues of rats given ifos had numerous histological irregularities. Seminiferous tubules were observed to be shrunken and disorganized. Moreover, the cytoplasmic vacuolation was also seen in Figure 8c. Pyknotic nuclei and vacuolation are also seen in Figure 8c. Testes of rats treated with ifos and morin 100 revealed a mild regularity in testis structure. Tubules of rats treated with ifos and morin 100 still had histological irregularities and mild vacuolation (Figure 8d). Tubules of rats treated with ifos and morin 200 regained their normal shape. Mild irregularities were observed in testis tissues (Figure 8e).

***Impacts of morin on immunohistochemical changes in testis tissue***

**Figure 9:**
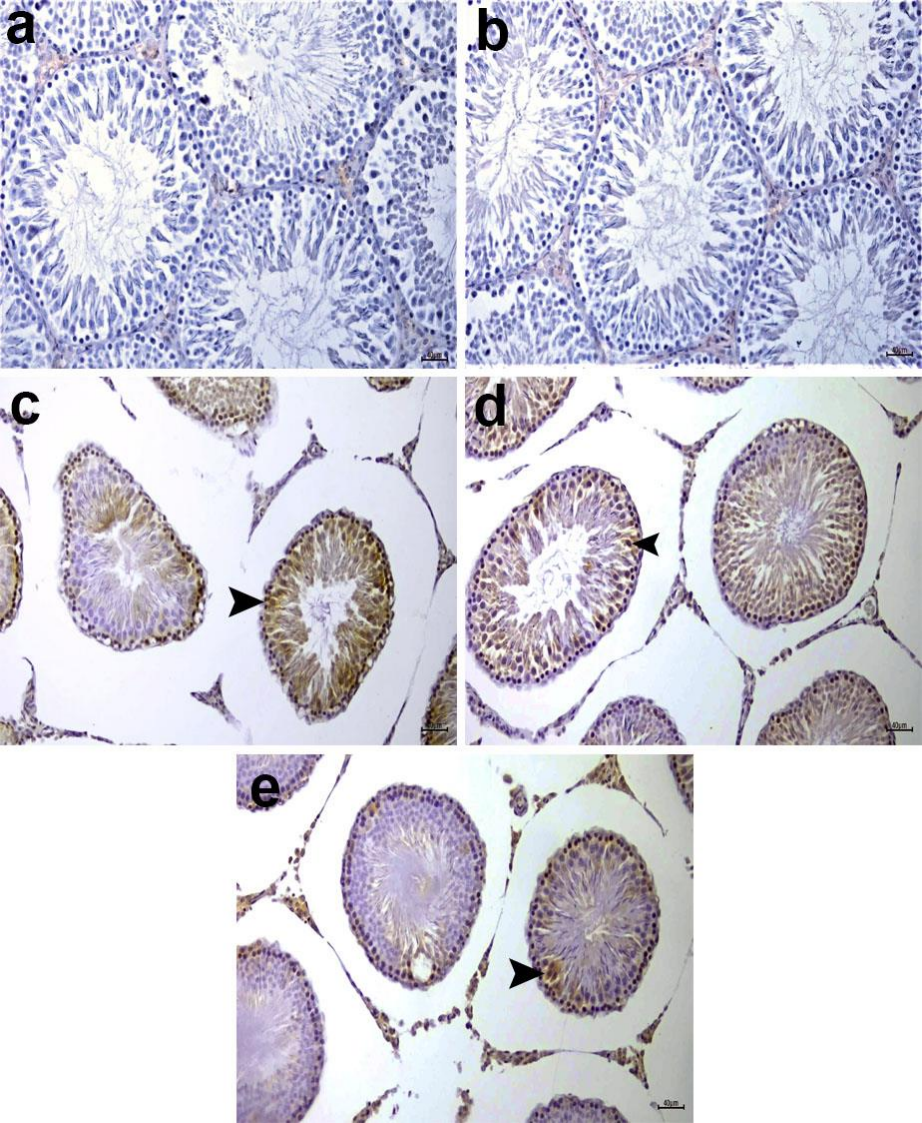
Control (a) and morin (b) groups do not show 8-OHdG immunopositivity in the rat testis tissues

Table 2 shows the immunohistochemical staining results. Figure 9 provides details of the immunopositivity of 8-OHdG. The control and morin groups have not revealed 8-OHdG immunopositivity in testis germ cells (Figures 9a and 9b). Intense 8-OHdG immunopositivity was observed in spermatogonium, in addition to primary and secondary spermatocytes within the seminiferous tubules in ifos-treated testis tissues (Figure 9c). On the other hand, 8-OHdG immunopositivity was decreased in the ifos+morin 100 group when compared with the ifos group, but a significant difference was not detected (Figure 9d, *P*>0.05). Immunohistochemical staining for 8-OHdG revealed mild immunopositivity in spermatogonium and primary and secondary spermatocytes in ifos+morin 200 treated rat testis tissues (Figure 9e). A significant difference was detected compared to the ifos+morin 100 group (*P*<0.05).

## Discussion

After the developments in cancer treatments that increase survival, there is an increase in the number of men who have or want to have children after chemotherapy. However, testicular damage is seen as a side effect of many of the drugs (29). On the other hand, studies to reduce testicular damage of chemotherapeutics are increasing, and positive results are obtained (18). IFOS is frequently used in the treatment of various malignancies, especially in children (1). Although there are various studies on the toxicity of IFOS (3, 5), testicular damage has not been sufficiently clarified. For this reason, it is essential to elucidate the mechanisms that play a role in testicular damage of IFOS and to find new substances that can protect against this damage. For this purpose, the effects of IFOS on testicular tissue were investigated through oxidative stress and some molecular pathways associated with oxidative stress. Moreover, it was examined whether morin has a potential protective effect on these pathways.

Oxidative stress is at the root of the toxicity mechanism of many compounds, including chemotherapeutic agents (12, 30). One of these agents is IFOS. IFOS is metabolized by CYP3A4 and the metabolites formed are highly toxic, unlike cyclophosphamide. These metabolites consist of highly electrophilic cytotoxic nitrogen mustards (iphosphoramide mustard or isophosphoramide mustard) and acrolein as well as chloroacetaldehyde (6). Isophosphoramide mustard, acrolein, and chloroacetaldehyde are strong consumers of GSH (6, 31). GSH provides important protection against oxidative damage by scavenging ROS in the organism (32, 33). It has been reported that the metabolites of IFOS consume GSH, resulting in oxidative damage in various tissues and lipid peroxidation (6). On the other hand, there is not enough information about the fate of SOD, CAT, and GPx enzymes, which are involved in anti-oxidant defense, after IFOS treatment. In the present study, the effects of IFOS and morin applications on oxidative stress in testicular tissue were evaluated by analysis of SOD, CAT, and GPx enzyme activities, and MDA and GSH levels. According to the data obtained, it was observed that IFOS, similar to the literature, depleted GSH stores as well as inhibiting the activities of enzymatic anti-oxidant enzymes, and, in parallel, an increase in lipid peroxidation occurred. Morin, on the other hand, alleviated oxidative stress by renewing GSH stores with its anti-oxidant property. In addition, it is understood from the decrease in MDA levels that it protects against lipid peroxidation by increasing SOD, CAT, and GPx activities. Previous studies have shown that oxidative stress caused by various toxic compounds can be alleviated by morin treatment and can be provided to protect against testicular tissue damage (18, 34).

Another factor that protects against oxidative stress is Nrf-2 (35). Transcription of phase-II detoxification enzymes, including HO-1, is triggered when Nrf-2 is separated from Keap1 and translocated to the nucleus (36-38). Various chemotherapeutic agents, including IFOS, have been reported to cause oxidative stress by suppressing Nrf-2 expression (35, 39). Studies investigating the relationship between IFOS and Nrf-2 in testicular tissue are insufficient. In the present study, it was revealed that IFOS suppressed Nrf-2 transcription in testicular tissue and, accordingly, the HO-1 gene was down-regulated. On the other hand, there is increasing evidence that plant-derived substances can activate the Nrf-2/HO-1 pathway (9, 40). In our study, we saw that plant-derived morin can trigger the Nrf-2/HO-1 pathway suppressed by IFOS in testicular tissue. 

FOXO, a subfamily of the Forkhead transcription factors family, regulates the expression of ROS detoxification enzymes such as catalase and SOD2, which reduce oxidative stress (41). In a previous study, it was stated that FOXO1 was significantly decreased in the testicular tissues of hamsters given Bisphenol S, while FOXO1 increased after melatonin treatment and oxidative stress was alleviated (42). In another study, hamsters were given sodium fluoride, and similar results were obtained (43). Consistent with the literature, in our study, it was observed that IFOS down-regulated FOXO1 expression, and morin treatment activates the expression of this gene.

Free radical formation can cause base modifications and strand breaks in DNA by oxidizing the guanine residues of 8-OHdG, an oxidized nucleoside of DNA. For this reason, 8-OHdG measurement is widely used in the determination of oxidative DNA damage (44). In a previous study, it was reported that acrylamide, which is a toxic agent, causes an increase in 8-OHdG levels together with oxidative stress (45). In the present study, it was observed that oxidative stress was triggered after IFOS administration, and 8-OHdG levels increased in testicular tissues, possibly due to this. On the other hand, it was determined that morin treatment could alleviate DNA damage by suppressing oxidative stress.

Although inflammation is an important mechanism for protection against various pathogens and regeneration after tissue injury, it causes tissue damage when it occurs in an uncontrolled manner (5, 46). The occurrence of inflammation in the testicular tissue has negative effects on reproduction (47). As stated in previous studies, oxidative stress can up-regulate the expression of pro-inflammatory cytokines by activation of signaling pathways such as NF-кB, thereby triggering the inflammatory response (5, 48). In a previous study, it was reported that IFOS inhibited SOD3, GPx1, and CAT activities in the bladder and caused an increase in TNF-α and iNOS levels in correlation with increased MDA levels. It has been stated that direct contact of acrolein with urinary bladder urothelium and increased ROS and RNS compounds may cause inflammation and damage to urinary bladder urothelium (49). It has been reported that IFOS also triggers inflammatory cytokines in the nervous system and causes neuroinflammation (31). In this study, it has been proven by both western blot and histopathological analyses that IFOS can cause damage by triggering inflammation in the testicular tissue. On the other hand, it has been shown that morin treatment can alleviate IFOS-induced testicular inflammation.

The ER performs a variety of roles to maintain cellular homeostasis as well as detoxify xenobiotics and maintain calcium homeostasis. On the other hand, the imbalance occurring in various physiological and pathological processes causes ER stress by causing the accumulation of unfolded or misfolded proteins in the ER lumen (50). Under ER stress, the unfolded protein response (UPR) is enhanced to maintain protein homeostasis. There are three different effectors in the UPR response. These are ATF-6, PERK, and IRE1 (51). However, the continuity of the UPR has harmful consequences such as apoptosis (52). Mitochondria-independent apoptosis is also involved as a result of CHOP activation (53). Recently, ER stress as the mechanism responsible for drug toxicities has been intensively investigated in addition to mitochondrial dysfunction and oxidative stress. In previous studies, it has been reported that various toxic agents cause an increase in ER stress markers in testicular tissue and this may have a negative effect on reproduction (54, 55). Similarly, in our study, it can be said that after IFOS application, ATF-6, PERK, IRE1, and GRP-78 expressions were triggered, the UPR response started, and the apoptotic process occurred with increased CHOP expression. Considering that there is a connection between ER stress and oxidative stress, it was thought that morin could be effective against ER stress in the study, and the results showed that cod could also indirectly alleviate ER stress by suppressing oxidative stress. Because after morin treatment, there was a remarkable decrease in mRNA transcript levels of ATF-6, PERK, IRE1, GRP-78, and CHOP genes in testicular tissue.

Apoptosis along with oxidative stress are important factors contributing to many conditions that cause infertility (56). The levels of Bax, an apoptotic protein, increase under oxidative stress conditions and translocate into the mitochondria of germ cells, impairing membrane permeability. This causes the release of cytochrome C from the mitochondria (57, 58). Cytochrome C, on the other hand, forms an apoptosome with apaf-1 and procaspase-9, and then caspase-3 is activated (58). Bcl-2, an anti-apoptotic protein, ensures cell survival by maintaining membrane integrity (57, 59). Previous studies have reported that various chemotherapeutic agents reduce fertility by activating the apoptotic pathway, but herbal-derived drugs have an anti-apoptotic effect and have a positive effect on fertility (57, 58). In our study, it was observed that IFOS caused apoptosis by triggering the apaf-1/caspase-3 pathway, while apoptosis was suppressed by morin treatment. Possibly, morin may have decreased Bax levels in association with its alleviation of oxidative stress and thus inhibited apaf-1 and caspase-3 activity. Moreover, morin may have improved mitochondrial membrane permeability by increasing mRNA transcript levels of the anti-apoptotic Bcl-2. 

It is known that the AKT signaling pathway plays an important role in cell growth and proliferation (60, 61). AKT signaling also makes an important contribution to proliferation in Leydig cells (62). There are three different isoforms of AKT: AKT1, AKT2, and AKT3 (60). In the present study, it was determined that IFOS down-regulated AKT2 expression. On the other hand, it was observed that AKT2 expression was triggered by morin treatment. A previous study reported that triphenyltin significantly reduced AKT1 and AKT2 phosphorylation and correlated well with reduced Leydig cell numbers (63).

Autophagy, which is a normal biological process, causes cellular dysfunction when it occurs at an advanced level (14). Beclin-1 is an important biomarker used to monitor autophagosome formation and autophagic pathway (64). Previous studies have reported that increased expression of beclin-1 in testicular tissue causes damage to testicular tissue and causes dysfunction (65, 66). In our study, a significant increase in the levels of beclin-1 protein was detected in testicular tissue of rats given IFOS. However, after morin treatment, beclin-1 levels were suppressed and protection against autophagy was provided.

**Table 1:** Sequences of primers of genes used in RT-PCR method

**Gene**	**Sequences (5’-3’)**	**Length (bp)**	**Accession No**
**Nrf2**	F: TTTGTAGATGACCATGAGTCGC R: TCCTGCCAAACTTGCTCCAT	161	NM_031789.2
**HO-1**	F: ATGTCCCAGGATTTGTCCGA R: ATGGTACAAGGAGGCCATCA	144	NM_012580.2
**Akt2**	F: GAGTACTTGCACTCGACGGA R: CCATGAGGATGAGCTCGAAG	304	NM_017093.1
**FOXO1**	F: CAGCCAGGCACCTCATAACA R: TCAAGCGGTTCATGGCAGAT	143	NM_001191846.3
**ATF-6**	F: TCAACTCAGCACGTTCCTGA R: GACCAGTGACAGGCTTCTCT	130	NM_001107196.1
**PERK**	F: GATGCCGAGAATCATGGGAA R: AGATTCGAGAAGGGACTCCA	198	NM_031599.2
**IRE1**	F: GCAGTTCCAGTACATTGCCATTG R: CAGGTCTCTGTGAACAATGTTGA	163	NM_001191926.1
**GRP78**	F: CATGCAGTTGTGACTGTACCAG R: CTCTTATCCAGGCCATATGCAA	143	NM_013083.2
**CHOP**	F: GAAGCCTGGTATGAGGATCT R: GAACTCTGACTGGAATCTGG	209	NM_001109986.1
**Bax**	F: TTTCATCCAGGATCGAGCAG R: AATCATCCTCTGCAGCTCCA	154	NM_017059.2
**Bcl-2**	F: GACTTTGCAGAGATGTCCAG R: TCAGGTACTCAGTCATCCAC	214	NM_016993.2
**Apaf-1**	F: ACCTGAGGTGTCAGGACC R: CCGTCGAGCATGAGCCAA	192	NM_023979.2
**Caspase-3**	F: ACTGGAATGTCAGCTCGCAA R: GCAGTAGTCGCCTCTGAAGA	270	NM_012922.2
**-Actin**	F: CAGCCTTCCTTCTTGGGTATG R: AGCTCAGTAACAGTCCGCCT	360	NM_031144.3

R: Reverse; F: Forward

All data were expressed as mean±SD (n=7, number of repeats=3). Statistical significance (Control vs others: *P<0.05, **P<0.01, ***P<0.001, IFOS vs others: ^#^*P<*0.05, ^##^*P<*0.01, ^###^*P<*0.001, IFOS+Morin 100 vs IFOS+Morin 200: ^✢^*P<*0.05, ^✢✢^*P<*0.01, ^✢✢✢^*P<*0.001) was analyzed using one way ANOVA

All data were expressed as mean±SD (n=7, number of repeats=3). Statistical significance (Control vs others: *P<0.05, **P<0.01, ***P<0.001, IFOS vs others: ^#^*P<*0.05,^ ##^*P<*0.01, ^###^*P<*0.001, IFOS+Morin 100 vs IFOS+Morin 200: ^✢^*P<*0.05, ^✢✢^*P<*0.01, ^✢✢✢^*P<*0.001) was analyzed using one way ANOVA

SOD: Superoxide dismutase; CAT: catalase; GPx: glutathione peroxidase; MDA: malondialdehyde; GSH: glutathione; IFOS: ifosfamide

All data were expressed as mean±SD (n=7, number of repeats=3). Statistical significance (Control vs others: *P<0.05, **P<0.01, ***P<0.001, IFOS vs others: ^#^*P<*0.05, ^##^*P<*0.01, ^###^*P<*0.001, IFOS+Morin 100 vs IFOS+Morin 200: ^✢^*P<*0.05, ^✢✢^*P<*0.01, ^✢✢✢^*P<*0.001) was analyzed using one way ANOVA

All data were expressed as mean±SD (n=7, number of repeats=3). Statistical significance (Control vs others: *P<0.05, **P<0.01, ***P<0.001, IFOS vs others: ^#^*P<*0.05, ^##^*P<*0.01, ^###^*P<*0.001, IFOS+Morin 100 vs IFOS+Morin 200: ^✢^*P<*0.05, ^✢✢^*P<*0.01, ^✢✢✢^*P<*0.001) was analyzed using one way ANOVA

All data were expressed as mean±SD (n=7, number of repeats=3). Statistical significance (Control vs others: *P<0.05, **P<0.01, ***P<0.001, IFOS vs others: ^#^*P<*0.05, ^##^*P<*0.01, ^###^*P<*0.001, IFOS+Morin 100 vs IFOS+Morin 200: ^✢^*P<*0.05, ^✢✢^*P<*0.01, ^✢✢✢^*P<*0.001) was analyzed using one way ANOVA

All data were expressed as mean±SD (n=7, number of repeats=3). Statistical significance (Control vs others: *P<0.05, **P<0.01, ***P<0.001, IFOS vs others: ^#^*P<*0.05, ^##^*P<*0.01, ^###^*P<*0.001, IFOS + Morin 100 vs IFOS+Morin 200: ^✢^*P<*0.05, ^✢✢^*P<*0.01, ^✢✢✢^*P<*0.001) was analyzed using one way ANOVA

All data were expressed as mean±SD (n=7, number of repeats=3). Statistical significance (Control vs others: *P<0.05, **P<0.01, ***P<0.001, IFOS vs others: ^#^*P<*0.05, ^##^*P<*0.01, ^###^*P<*0.001, IFOS+Morin 100 vs IFOS+Morin 200: ^✢^*P<*0.05, ^✢✢^*P<*0.01, ^✢✢✢^*P<*0.001) was analyzed using one way ANOVA

Control (a) and morin (b) groups show normal histological architecture, spermatogonia (Sg), primary spermatocytes (ps), Leydig cells (Ic), spermatids (sd), and sperm cells (sp). Ifos group (c) draws attention with presence of irregular and disorganized structure, pyknosis (pyk), and cytoplasmic vacuolation (vc). Ifos+ morin 100 group (d) demonstrates a mild decrease in the intensity of disorganized structure and cytoplasmic vacuolation. Ifos +morin 200 group (e) demonstrates an obvious decrease in the intensity of disorganized structure. HE; x 40µm

Ifosfamide (IFOS) group (c) markedly increased 8-OHdG immunopositivity (arrowhead) in many spermatogonia and primary spermatocytes. Ifos+morin 100 group (d) shows mild decrease in 8-OHdG immunopositivity (arrowhead) in spermatogonia and primary spermatocytes. Ifos+morin 200 group (e) shows markedly decreased 8-OHdG immunopositivity (arrowhead) in spermatogonia and primary spermatocytes. IHC; x 40µm

**Table 2:** Immunohistochemical evaluation of 8-OHdG immunopositivity in the rat testis tissues

**Groups/Parameter **	**8-OHdG immunopositivity**
**Control**	0.28±0.18^a^
**Morin**	0.14±0.14^a^
**Ifos**	2.57±0.29^b^
**Ifos+Morin 100 **	2.28±0.47^b^
**Ifos+Morin 200**	1.00±0.30^c^

Values are expressed as mean±SEM of seven rats in each group. Different superscripts (a-c) in the same row indicate significant differences among groups (*P<*0.05)

## Conclusion

Taken together, it was determined that IFOS treatment in rats caused testicular damage by triggering oxidative stress, ER stress, inflammation, autophagy, and apoptosis in testicular tissues, resulting in histological irregularities. However, morin reduced oxidative stress by showing an anti-oxidant effect and thus, it also interrupted the reactions triggered by oxidative stress. Histopathological results also confirmed that morin protects testicular tissue against IFOS.

## Authors’ Contributions

All authors contributed to the study’s conception and design. F C, S K, C G, S C, M I, and FM K performed material preparation, data collection, and analysis. C G wrote the first draft of the manuscript, and all authors commented on previous versions of the manuscript. All authors read and approved the final manuscript.

## Conflicts of Interest

The authors declare no conflicts of interest.
